# A Naturally Occurring Canine Model of Autosomal Recessive Congenital Stationary Night Blindness

**DOI:** 10.1371/journal.pone.0137072

**Published:** 2015-09-14

**Authors:** Mineo Kondo, Gautami Das, Ryoetsu Imai, Evelyn Santana, Tomio Nakashita, Miho Imawaka, Kosuke Ueda, Hirohiko Ohtsuka, Kazuhiko Sakai, Takehiro Aihara, Kumiko Kato, Masahiko Sugimoto, Shinji Ueno, Yuji Nishizawa, Gustavo D. Aguirre, Keiko Miyadera

**Affiliations:** 1 Department of Ophthalmology, Mie University Graduate School of Medicine, Tsu, Japan; 2 School of Veterinary Medicine, University of Pennsylvania, Philadelphia, PA, United States of America; 3 Pharmaceutical Research Division, Takeda Pharmaceutical Co., Ltd., Fujisawa, Japan; 4 Kitayama Labes Co., Ltd., Ina, Japan; 5 Department of Ophthalmology, Nagoya University Graduate School of Medicine, Nagoya, Japan; 6 Department of Biomedical Sciences, Chubu University, Kasugai, Japan; Tohoku University, JAPAN

## Abstract

Congenital stationary night blindness (CSNB) is a non-progressive, clinically and genetically heterogeneous disease of impaired night vision. We report a naturally-occurring, stationary, autosomal recessive phenotype in beagle dogs with normal daylight vision but absent night vision. Affected dogs had normal retinas on clinical examination, but showed no detectable rod responses. They had “negative-type” mixed rod and cone responses in full-field ERGs. Their photopic long-flash ERGs had normal OFF-responses associated with severely reduced ON-responses. The phenotype is similar to the Schubert-Bornschein form of complete CSNB in humans. Homozygosity mapping ruled out most known CSNB candidates as well as *CACNA2D4* and *GNB3*. Three remaining genes were excluded based on sequencing the open reading frame and intron-exon boundaries (*RHO*, *NYX*), causal to a different form of CSNB (*RHO*) or X-chromosome (*NYX*, *CACNA1F*) location. Among the genes expressed in the photoreceptors and their synaptic terminals, and mGluR6 cascade and modulators, reduced expression of *GNAT1*, *CACNA2D4* and *NYX* was observed by qRT-PCR in both carrier (n = 2) and affected (n = 2) retinas whereas *CACNA1F* was down-regulated only in the affecteds. Retinal morphology revealed normal cellular layers and structure, and electron microscopy showed normal rod spherules and synaptic ribbons. No difference from normal was observed by immunohistochemistry (IHC) for antibodies labeling rods, cones and their presynaptic terminals. None of the retinas showed any sign of stress. Selected proteins of mGluR6 cascade and its modulators were examined by IHC and showed that PKCα weakly labeled the rod bipolar somata in the affected, but intensely labeled axonal terminals that appeared thickened and irregular. Dendritic terminals of ON-bipolar cells showed increased Goα labeling. Both PKCα and Goα labeled the more prominent bipolar dendrites that extended into the OPL in affected but not normal retinas. Interestingly, RGS11 showed no labeling in the affected retina. Our results indicate involvement of a yet unknown gene in this canine model of complete CSNB.

## Introduction

Vertebrate retinas have highly sensitive rods and less sensitive yet faster responding cones as the primary photoreceptors to detect light energy. The processing of this visual information into neural signals includes secondary (bipolar cells and horizontal processes) and tertiary retinal neurons (amacrine and retinal ganglion cells (RGCs)) before being transmitted to the brain, finally producing image-forming vision. While rods are for night vision, cones aid in daylight and color vision. In dark, the photoreceptors are depolarized and their membrane potential becomes more positive as glutamate is continuously released into synapses with bipolar cells. The neurotransmitter binds to metabotropic glutamate receptor (mGluR6, encoded by *GRM6*) [1,2] leading to the activation of the alpha subunit of the G-protein coupled receptor Goα [3,4] thereby causing the closure of the non-selective cation channel TRPM1. Upon light stimulation, the photoreceptors are hyperpolarized and their membrane potential becomes more negative as glutamate release is suppressed.

Visual information is channeled through two different pathways in bipolar cells; ON-bipolars express either ionotropic (iGluR) or metabotropic (mGluR6) glutamate receptors, while OFF bipolar cells express only the ionotropic (AMPA/ kainate) glutamate receptors [1,2,5–7]. The rods synapse with a single class of rod bipolar (RB) cells that are all depolarizing (ON), while cones can synapse with 9 different cone bipolars (CBs) which can be either depolarizing (ON) or hyperpolarizing (OFF) [8–11]. Unlike the CB cells, RB cells cannot directly contact the RGC. Instead, the AII amacrine cells relay the rod signal to RGCs, the primary rod pathway with highest sensitivity. At high scotopic intensity, rods make electrical coupling with cones via gap junctions between rod and cone pedicles to form either excitatory synapse with ON-CB cells or inhibitory synapse with OFF-CB cells [12,13]. This is a secondary pathway with intermediate sensitivity. In addition, a tertiary pathway with low sensitivity exists where rods can directly contact the OFF-CB cells [12]. Disruption at any step of the signal transduction in the retina can lead to vision defects.

Retinal function can be assessed objectively by electroretinography (ERG) which represents a complex response from different retinal cells [14] due to changes in the polarization of the photoreceptors and bipolar cells following photic stimulation. Under dark-adapted (scotopic) conditions, ERG reflects the rod-driven circuitry while the cone-driven one can be obtained under light-adaptation (photopic). In a dark-adapted retina, the ERG response to a very brief dim flash of light generates an initial cornea negative a-wave due to the hyperpolarization of the photoreceptors followed by the positive b-wave that originates from the depolarizing bipolar cells [14].

Night blindness occurs as an early sign of various retinal dystrophies where there is dysfunction and/or degeneration of photoreceptors, or from defects in ON-bipolar cells caused by genetic defects. Unlike night blindness where the photoreceptors degenerate and the symptoms are progressive, congenital stationary night blindness (CSNB) is a non-progressive, clinically and genetically heterogeneous disease of impaired night vision, often accompanied by decreased visual acuity and refractive errors in human patients [15]. Based on ERG pattern it is clinically subdivided into Riggs and Schubert-Bornschein types. The Riggs form is characterized by reduced a-waves as the defect lies in the photoreceptors [16]. The Schubert-Bornschein form, on the other hand, shows “negative-type” ERG with a normal amplitude a-wave and a smaller, significantly reduced b-wave [17]. Here the defect lies in signaling from photoreceptors to bipolar cells, or intrinsic to bipolar cells. The Schubert-Bornschein form can be further classified [18] into: (i) complete type (cCSNB) where the b-wave response is drastically reduced; and (ii) incomplete type (icCSNB) where signal transmission from photoreceptors to the bipolar cells is partially blocked, as both the ON-OFF pathways are affected and patients have reduced but still some recordable residual rod function [15].

There is great genetic and allelic heterogeneity in CSNB. For the Schubert-Bornschein form, mutations in *GRM6*, *TRPM1*, *GPR179* and *LRIT3* cause autosomal recessive cCSNB in patients [19–24], while *NYX* is responsible for X-linked cCSNB [25,26]. All these genes encode proteins that are expressed in the dendritic tips of the bipolar cells and are involved in the ON-bipolar mGluR6 cascade. For icCSNB, mutations in *CABP4*, a neuronal calcium binding protein in the photoreceptor synaptic terminal, cause autosomal recessive disease [27], while *CACNA1F*, encoding the alpha 1f subunit of Cav1.4 calcium channel and localized in the photoreceptor ribbon synapse active zone [28] causes X-linked icCSNB [29,30]. Riggs disease, on the other hand, is caused by mutations in *RHO* and *PDE6B -* both involved in phototransduction [31,32]—while *SLC24A1*, a sodium-calcium exchanger in the rod outer segment (ROS) is responsible for autosomal recessive disease [33]. *GNAT1*, another gene in the phototransduction pathway, can cause both the forms of CSNB [34,35].

Animal models of human disease play an important role in studying disease mechanisms and development of new treatments. Various naturally occurring and genetically manipulated animal models of CSNB have been identified; to date there are 7 mouse [22,36–42] and a horse model [43,44] for cCSNB. Herein, we report a naturally occurring disease in the beagle dog that is a model for autosomal recessive cCSNB in man. Our clinical, genetic, molecular and morphological findings suggest a yet unknown gene that impairs the signal transduction in the ON-bipolar cells. For translational applications, such a model is particularly important because the similarities between the canine and human eyes facilitates surgical approaches and non-invasive assessment methods that make it easier to develop and test new treatments [45,46].

## Materials and Methods

### Ethics statement

The research was conducted in full compliance and strict accordance with the Association for Research in Vision and Ophthalmology (ARVO) Resolution on the Use of Animals in Ophthalmic and Vision Research. The protocol was approved by Takeda Pharmaceutical Company Ltd. (TEACUC-1322), Mie University Graduate School of Medicine (number: 24–49) Institutional Animal Care and Use Committee (IACUC), and University of Pennsylvania (number: 801870) IACUC. Even though none of the procedures used caused pain, all efforts were made to minimize dog suffering.

### Animals

**Fig 1:**
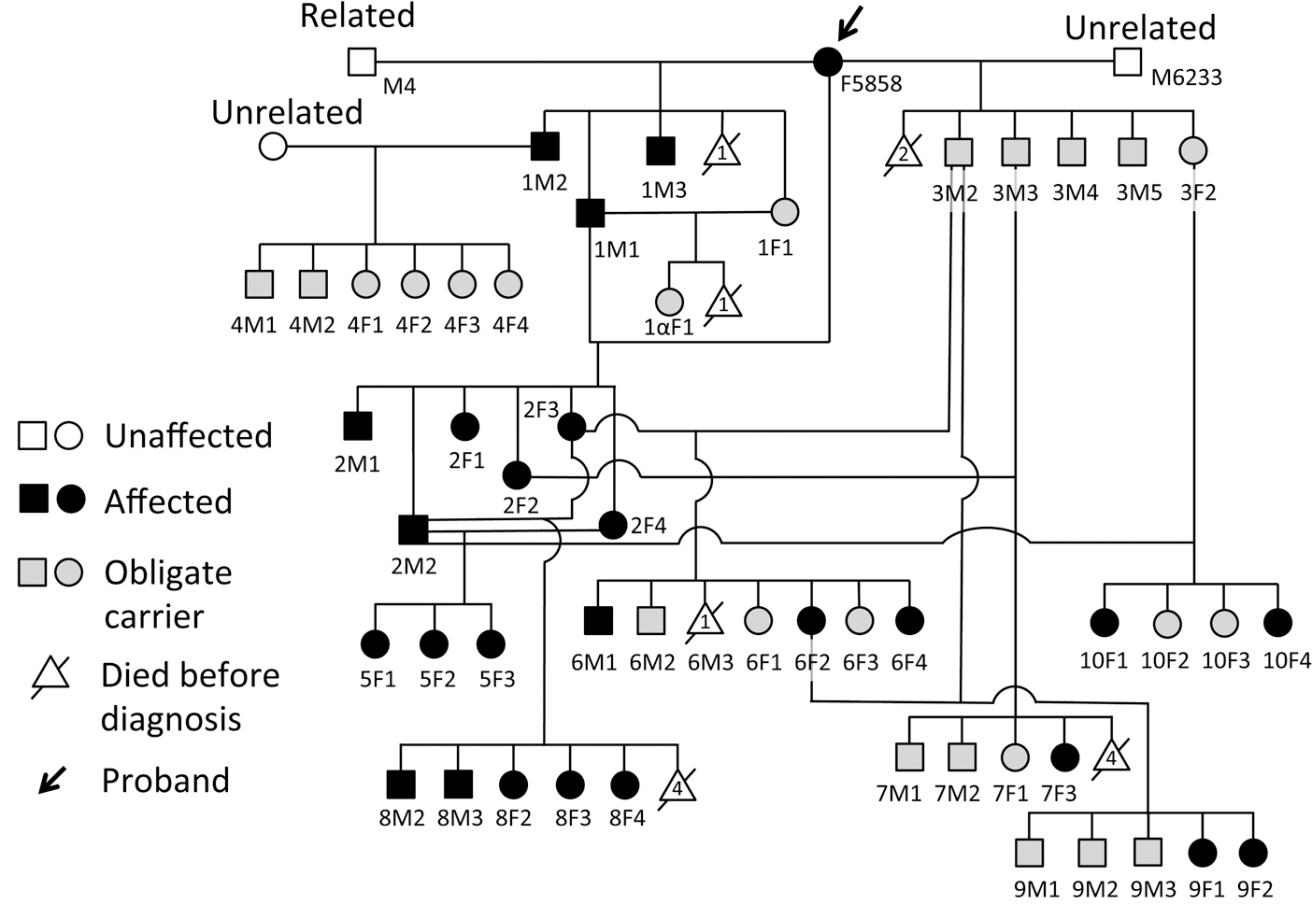
CSNB colony pedigree. Three beagle dogs, F5858, M4 and M6233, were used as founders for the CSNB colony that was then expanded by outcrossing, backcrossing and intercrossing. Breeding affected to obligate heterozygous animals resulted in non-affected and affected progeny, while affected to affected matings always produced affected progeny. The disease is inherited as an autosomal recessive trait.

Sixty beagle dogs from one pedigree were studied. The proband dog, which was originally purchased from Japan Laboratory Animals, Inc. (Tokyo, Japan), was identified with vision difficulties during ophthalmic testing at Takeda Pharmaceutical Co. Ltd. in Japan after ERG abnormalities were first noted. To determine the inheritance pattern and develop a colony for research, we carried out outcross, intercross and backcross matings between affected, normal and putative carrier dogs (Fig 1). In addition, five normal beagle dogs, purchased from Kitayama Labes Company Ltd., were used as control for ERG and electron microscopy studies.

All dogs were housed individually in stainless steel cages (4.6 m^2^). The temperature of the rooms was maintained at 15° to 25°C and the lighting was on a 12-hour light/12-hour dark cycle. The animal facilities were at the Institute of Laboratory Animals of Takeda Pharmaceutical Company Ltd., Kitayama Labes Company Ltd. or at Mie University in Japan. Each animal was fed standard dog food and provided tap water. The cages were washed daily. All facilities had special wide indoor areas that allowed the dogs to exercise and interact with other dogs regularly in pairs or in larger groups. All of the dogs were housed in Japan and not at the University of Pennsylvania. Only the DNA and retinal tissues of specific samples were sent to the University of Pennsylvania for genetic, expression and immunohistochemical (IHC) analyses.

During the electroretinographic (ERG) experiments, the dogs were anesthetized by intramuscular injections of ketamine (11 mg/kg, IM) and xylazine (2 mg/kg, IM). Xylazine was infused continuously for three hours to minimize animal suffering and for analgesia.

### Ophthalmic examinations

Ophthalmic examinations were conducted every six months after birth. Examinations of cornea, anterior chamber, iris, and lens were performed by slit-lamp biomicroscopy. The vitreous and retina were examined by indirect ophthalmoscopy. A fundus camera (Genesis, Kowa Co., Nagoya, Japan) was used for fundus photography and fluorescein angiography.

### Electroretinography

Following dark-adaptation for 30 min, animals were anesthetized with xylazine (2 mg/kg, IM) and ketamine (11 mg/kg, IM). ERGs were then recorded with a contact lens electrode having white light-emitting diodes (LEDs) built into the electrode [47]. Twelve stimuli intensity steps ranging from -4.0 to 1.5 log cd-s/m^2^ (photopic units) were used for the scotopic ERGs, and four steps of stimuli ranging from -1.5 to 1.5 log cd-s/m^2^ were used for the photopic ERGs. The photopic ERGs were recorded on a rod-suppressing white background of 30 cd/m^2^. The signals were amplified, band pass filtered between 1 and 1000 Hz, and averaged by a computer-assisted signal analysis system (MEB-9100 Neuropack; Nihon Kohden, Tokyo, Japan). To study the retinal function of the ON- and OFF-pathways, photopic long-flash ERGs were also recorded using 200 msec white stimuli of 400 cd/m^2^ on a rod-suppressing white background of 30 cd/m^2^.

### Behavioral test of visual function

To confirm that the dogs had difficulties in seeing in the dark, we observed and recorded with an infrared video camera (DRV-PC1, Sony, Japan) their visual function through an obstacle course under two different ambient light levels of dim and bright light conditions.

### CSNB candidate gene analysis

#### PCR amplification and sequencing

DNA was isolated and PCR reactions were performed by standard methods [48]. Five μl of the amplicons were treated with 2μl USB ExoSAP-IT reagent (Affymetrix, Santa Clara, CA, USA) to remove unconsumed dNTPs and remaining primers and the products were then bi-directionally sequenced.

#### Marker selection and homozygosity haplotype analyses

To perform haplotype analyses by homozygosity mapping, we genotyped selected SNPs and microsatellite repeat markers, both within and flanking the known CSNB candidate genes viz. *TRPM1*, *GRM6*, *GPR179*, *LRIT3*, *SLC24A1*, *GNAT1*, *CABP4*, *CACNA1F*, *NYX*, *RHO* and *PDE6B* for their potential association with the disease. In addition, we included *CACNA2D4* as a heterozygous mutation (p.Arg818Cys) has been reported in exon 25 of this gene in a patient with icCSNB although the functional implication of the variant is unknown [49]. GNB3, expressed in the ON-bipolar cells, forms the G protein heterotrimer and couples mGluR6 to TRPM1 [50] also was included in the analysis. S1 Table lists all the markers analyzed in the study.

For all but 1 gene (*CACNA2D4*), marker selection was based on the canine genome assembly CanFam 3.1. As *CACNA2D4* was not included in the CanFam 3.1 release, but was listed in CanFam 2.0, we have listed all the genes and markers used for homozygosity mapping according to CanFam 2.0 coordinates (S1 Table). Depending on the gene, ~6–15 markers were selected per gene for typing; 1 flanking on either side of the gene while the remaining were intragenic. For *LRIT3*, *GNB3*, *GNAT1*, *RHO* and *NYX* the initial set of markers studied were uninformative. Hence the entire coding region of these genes as well as their intron-exon boundaries were sequenced. New informative markers thus identified were used in haplotype analyses except for *RHO* and *NYX*. Those markers not listed in CanFam 3.1 are identified as 'Novel' in S1 Table.

### RNA extraction, cDNA synthesis and quantitative real-time PCR analysis

Whole retinas were isolated from the eyecups within 1–2 min of death and flash frozen in liquid nitrogen. They were stored at −80°C until used. RNA was extracted from retinal tissues of 2 affected (13, 25 mon) and 2 carrier (13, 25 mon) female beagles from the CSNB study colony. Three unrelated normal mixed-breed dogs (2 females, 1 male, ages = 7.5–14 mon) were used as controls. The RNA samples were DNase treated and reverse-transcribed as described previously [48]. Quantitative Real-time PCR (qRT-PCR) was done using 25 ng cDNA in a total volume of 25 μl in 96-well microwell plates on the Applied Biosystems 7500 Real-Time PCR System (Applied Biosystems, Foster City, CA, USA). For each sample, 3 technical replicates were run. S2 Table provides details and lists all the qRT-PCR primers used in the study. Fold change was calculated by the ∆∆CT method [51]. We considered any gene to be differentially expressed if both the samples in a group had a fold change ≥±2.

### Tissue processing, histology and immunohistochemistry

Tissues were collected from selected dogs after euthanasia using an overdose of intravenous euthanasia solution (Euthasol; Virbac, Fort Worth, TX, USA). Eyes from 4 beagle dogs from the CSNB colony in Japan (2 affected and 2 carriers) were used for morphologic and IHC studies; these represent the fellow eyes of the dogs used for the gene expression studies. Four unrelated dogs from the US were used: 3 beagles (ages 7 mon, 3.5 and 4.3 yrs) and a Labrador-greyhound mixed breed (12 mon) were used as normal controls. Processing of the eyes for IHC was done using two protocols. In the standard protocol [52], the enucleated eyes were fixed in 4% paraformaldehyde (PF) at 4°C for 1.5 hours. The anterior segment and vitreous from the posterior segment at ora serrata were removed and the posterior segment was kept at 4°C for another 1.5 hours; tissues were transferred to 2% PF for 24 hrs and trimmed into rectangles extending from the optic disc to the ora serrata along the superior and inferior meridians. The tissues were then incubated for 24 hrs in each of 15% and 30% sucrose, and finally embedded in Tissue-Tek O.C.T (Optimal Cutting Temperature) compound (Sakura Finetek, Torrence, CA, USA). This procedure was done in 2 normal controls.

In parallel with other ongoing studies, we found that expression of most bipolar cell markers was drastically reduced when the 2% PF stage of fixation was extended to 48 hrs or longer; this was the case of the samples from Japan that were shipped to the University of Pennsylvania in 2% PF. These samples came in two different shipments- retinas from 6F4 (affected) and 7F1 (carrier) were in 2% PF for 48 hours whereas shipment for 5F1 (affected) and 6F1 (carrier) was delayed in transit and hence they were in 2% PF for ~5–6 days. We found no differences in immunolabeling of samples at these two time points. To address this issue, 2 beagle controls were processed so that the trimmed tissues at the 2% PF step of the procedure varied between 24 hrs (standard), 36 and 48 hrs. All subsequent steps of the procedure were the same.

Ten μm retinal cryosections were stained with hematoxylin and eosin (H&E) or used for morphology and IHC. For each dog (4 control, 2 carrier and 2 affected), a section from the superior quadrant was used for quantitative evaluation of the outer (ONL) and inner (INL) nuclear layer thickness (μm) and cell counts (rows of nuclei). Beginning 1000μm from the ora serrata, and extending centrally in 1000μm intervals, the number of rows of nuclei and thickness of the ONL and INL were determined in three areas of a 40X field and averaged.

For IHC, the cryosections were incubated overnight with appropriate primary antibodies (see S3 Table for details) dissolved in dilution buffer (0.025% Triton X-100, 0.5% sodium azide and 1.5% normal goat serum in PBS) after a blocking step with 10% normal goat serum. For hCAR, L/M and S opsin, BSA was used instead of normal goat serum. Since some of the antibodies were poorly labeling the bipolar cells as well as photoreceptor terminals, an antigen retrieval step was used prior to immunolabeling the samples. For this, tissues were incubated in TBST (1x TBS/0.1% Tween 20/ 0.02 sodium azide) for 5 min. Fifty ml 1x Antigen Unmasking Solution, Low pH (Vector Laboratories Inc, California, USA) was added to a coplin jar containing the tissue slides and transferred to a decloaking chamber (Biocare Medical, Concord, CA, USA) at 125°C for 90 sec (pressure 25 lb) followed by 90°C for 10 seconds. Tissues were next incubated with 1X TBST for 10 min and then washed with 1X PBS for 5 min each. In all cases, antigen-antibody complexes were visualized with fluorochrome-labeled secondary antibodies (Alexa Fluor Dyes, Molecular Probes, Invitrogen, Carlsbad, CA) with 1:200 dilution and DAPI (4´,6-diamidino-2-phenylindole) used to stain cell nuclei. For LRIT3, TRPM1, mGluR6 and CtBP2 the secondary antibodies were diluted at 1:300. Slides were mounted with a medium composed of polyvinyl alcohol and DABCO (1,4 diazobizyklo-[2.2.2]oktan) (Gelvatol; Sigma-Aldrich, St. Louis, MO, USA) and then examined under 20X as well as 40X objective on an epifluorescence microscope (Axioplan; Carl Zeiss Meditec, Oberkochen, Germany). Images were digitally photographed from the same retinal regions, ~7,500 μm central to the ora serrata, and which were representative of the immunolabeling results for that eye. A Spot 4.0 camera (Diagnostic Instruments, Inc.) was used and settings were constant in all animals for each antibody used.

### Electron microscopy

Eyes were collected from 2-year-old normal and CSNB dogs after euthanasia. Following removal of anterior segment, the retinas were fixed in 2.5% glutaraldehyde for 2 hrs. They were subsequently fixed in 1% osmium tetroxide for 90 min, and then dehydrated through a graded series of ethanol (50–100%), and cleared in propylene oxide. Finally, the tissues were embedded in epoxy resin. Ultrathin sections were cut on an ultramicrotome (Ultracut E; Reichert-Jung, Vienna, Austria) and stained with uranyl acetate and lead citrate. The stained sections were observed by transmission electron microscopy (JEM-1400EX; JEOL Ltd., Tokyo, Japan).

## Results

### Autosomal recessive inheritance for CSNB

To define the inheritance pattern for the disease, a colony of beagle dogs with CSNB was developed from 3 founder animals (F5858, M4 and M6233), and expanded by outcross, backcross and intercross to produce affected (n = 24) and obligate carrier (n = 22) dogs (Fig 1). Dominant inheritance was excluded as the progeny (n = 11), both male and female, were phenotypically normal when affected dogs were outcrossed to unrelated normal dogs (expected = 5-6/11 dogs affected; observed = 0/11). When the progeny was used in backcrosses to affected animals, they produced affected and non-affected offspring in approximately 1:1 ratio as expected for an autosomal recessive disease. X-linked inheritance was ruled out as both sexes were affected in the pedigree. Eight of the nineteen offspring produced as a result of mating between obligate carrier and affected dogs had the CSNB phenotype. Mating between affected dogs resulted in pups all of which had the CSNB phenotype. These results are consistent with autosomal recessive mode of inheritance.

### Clinical observations and non-progressive nature of the disease

**Fig 2:**
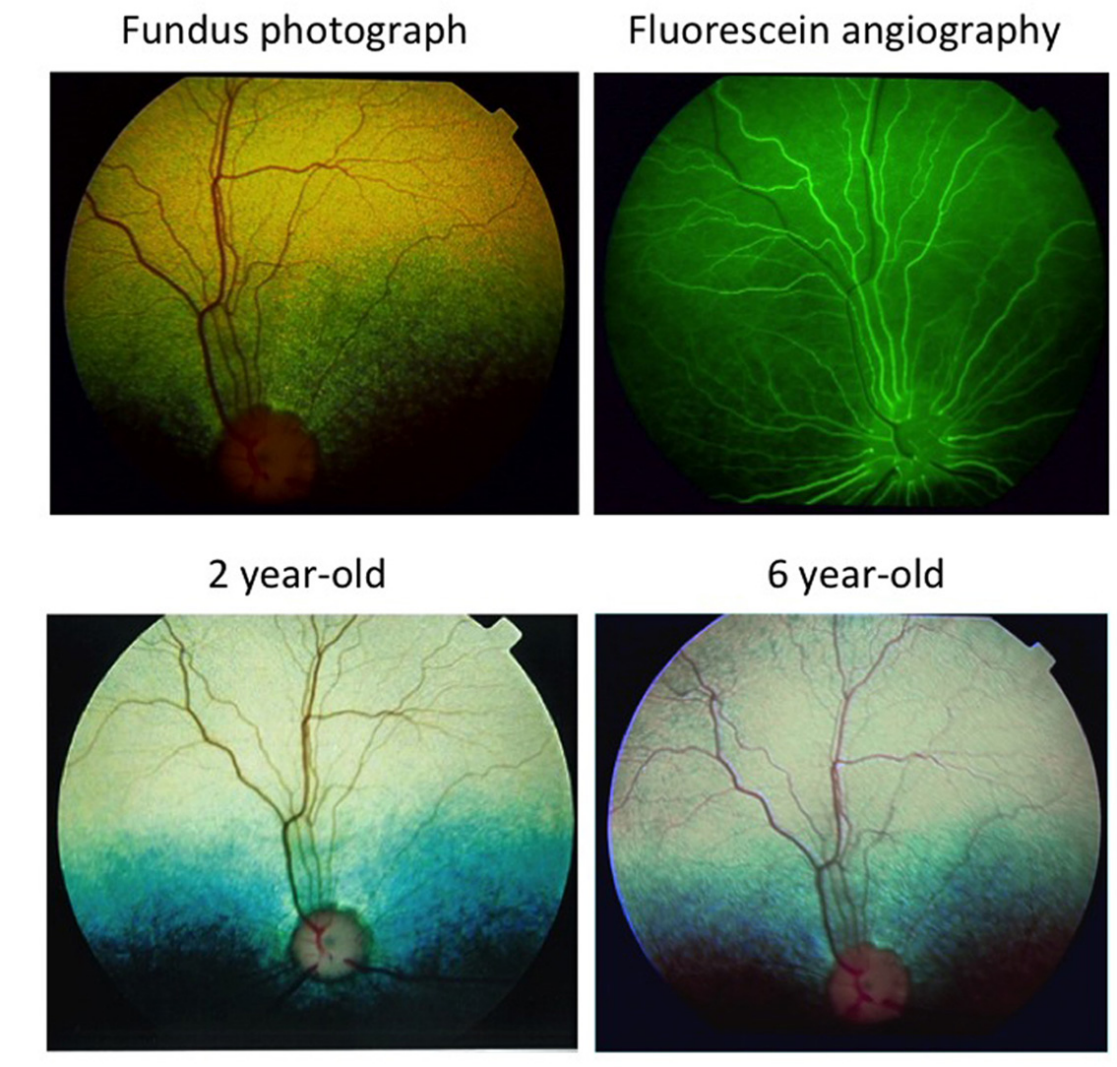
Retinal examination shows no abnormalities over time. Fundus photographs of the same dog show normal retinal integrity and vasculature. The tapetal retina (green-yellow color above the optic disc) shows a different color in the photographs due to the different light intensities used. The retina remains normal and unchanged during a 4 year observation period. The fluorescein angiogram (top right) shows normal vascular perfusion during the late arteriolar phase; the venules are just beginning to fill with contrast.

CSNB affected dogs had normal cornea, anterior chamber, and clear lens. Their fundus and fluorescein angiogram were normal (Fig 2, upper trace). The stationary nature of the disease in these dogs was evident from the fundus pictures taken at 2 different time points 4 years apart. Fig 2, lower trace is a representative fundus picture of the same dog (#5858) at 6 years of age showing no apparent difference with the one taken at 2 years of age.

We tested visual function under bright and dim light conditions. The CSNB affected dogs could easily maneuver through an obstacle course under bright light; however, they collided with obstacles at low illumination of light. In this reduced light, they walked slowly, rising their forelimbs high (S1 Movie) emphasizing their difficulty in night vision.

### Defect in signal transmission from photoreceptors to ON-bipolar cells

**Fig 3:**
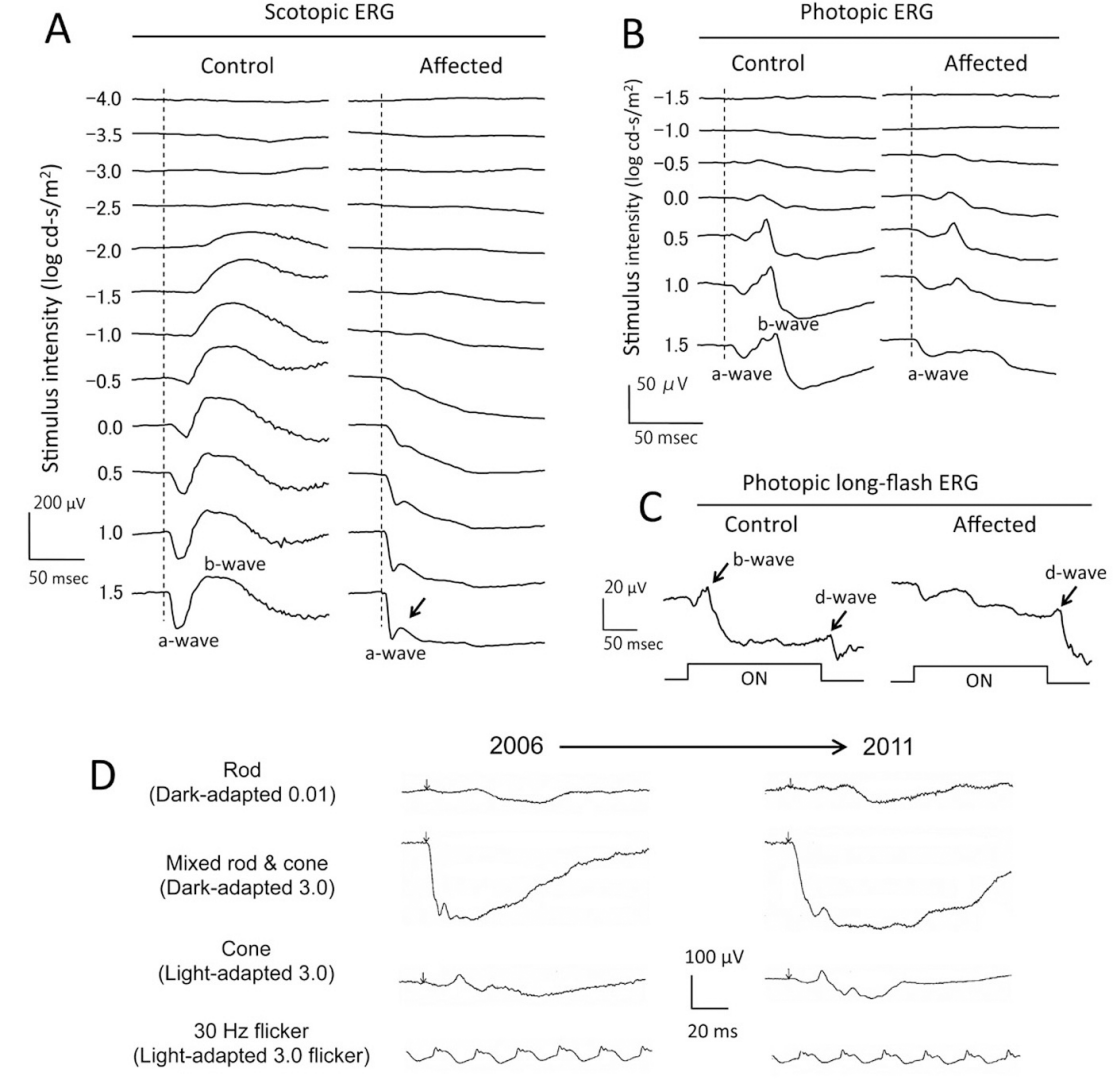
Scotopic and photopic ERGs show a defect in signal transmission from rods and cones to ON-bipolar cells. Electroretinograms (ERGs) recorded from CSNB dogs. **A**) Dark-adapted (scotopic) ERGs elicited by different stimulus intensities. Affected dog shows normal a-wave but loss of the b-wave. **B**) Light-adapted (photopic) ERGs elicited by different stimulus intensities. Affected dog shows a reduction of the b-wave at higher stimulus intensities of 1.0–1.5 log cd-s/m^2^. **C**) Photopic long-flash ERG using 200 ms stimuli of 400 cd/m^2^. Affected dog shows reduced ON-response (b-wave), but normal OFF-response (d-wave). **D**) Standard ERGs recommended by ISCEV [70] recorded from the same affected dog at 2 and 7years-of-age showed no progressive ERG changes in the 5 year interval.

Scotopic and photopic ERG analysis was used to assess retinal function in normal, carrier and affected dogs. The dark-adapted (scotopic) ERGs elicited by different intensities of stimuli in normal and affected dogs are shown in Fig 3A. The amplitudes of the a-waves, originating from the photoreceptors, were approximately equal for both the phenotypes; in contrast, the dark-adapted b-waves, originating primarily from the rod ON-bipolar cells, were absent at lower intensities. At higher intensities, 0.5 to 1.5 log cd-s/m^2^, a very small positive inflection was seen in the affected ERG (Fig 3A, arrow). At the intermediate to higher intensities, the ERG waveforms had a “negative-type” shape, and the amplitude of the b-wave was much reduced and smaller than the a-wave. Similar to the scotopic ERGs, the amplitudes of the a-wave under light-adapted, photopic conditions were nearly equal in both the groups. The b-waves of affected dogs were nearly the same as those of normal at moderate light intensities of 0 to 0.5 log cd-s/m^2^, but were severely reduced at higher intensities of 1.0 to 1.5 log cd-s/m^2^ (Fig 3B). To study the retinal function of the post-receptoral ON- and OFF-pathways, we next recorded the photopic ERGs with long duration stimuli of 200 msec (Fig 3C). The ON-response (b-wave) was markedly reduced associated with normal OFF-responses (d-wave) in the affected dogs. These ERG findings did not change in the 5 year interval (Fig 3D). The ERG findings in obligate heterozygous dogs were the same as for genetically normal dogs. Taken together, our ERG data indicate a defect in the signal transmission from both rod and cone photoreceptors to ON-bipolar cells and is consistent with complete CSNB of the Schubert-Bornschein type.

### Exclusion of known CSNB candidate genes

**Fig 4:**
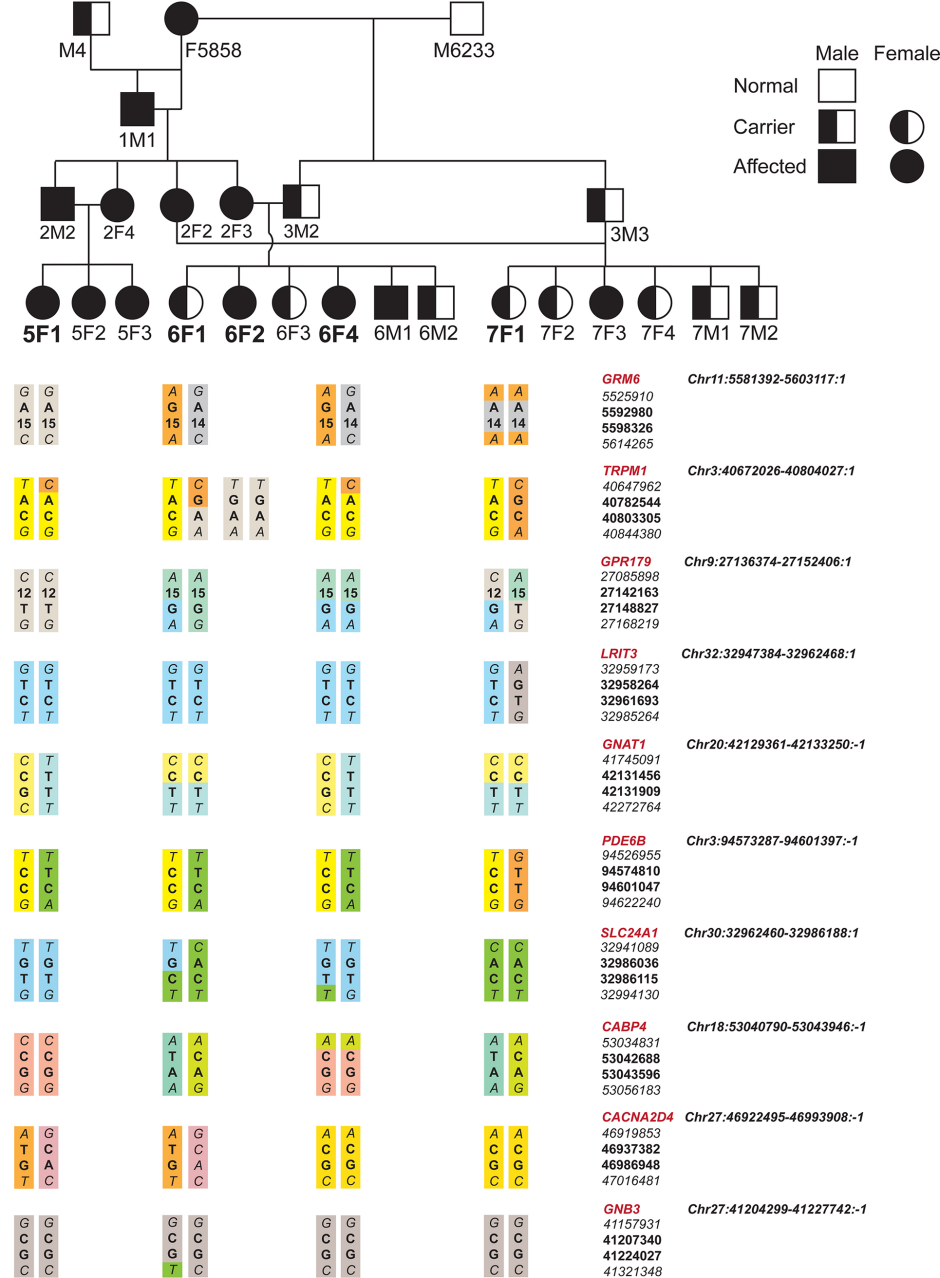
Haplotype ana lyses of candidate genes in CSNB pedigree. CSNB informative pedigree and haplotypes for the candidate genes studied. This pedigree is showing a subset of the beagle colony used for molecular genetic study. Phenotypic status was assigned by ERG and vision testing. Haplotypes for those genes with informative SNPs and microsatellites are shown in 5 representative animals out of the 10 samples examined (note that for affected animal 6F2 only *TRPM1* is illustrated as it shows a different haplotype from the other affecteds in the figure). Only 4 markers for each gene are shown to illustrate exclusion of candidate gene from disease. Markers in bold are intragenic, and flanking markers at either end of the haplotype block are in italics. See S1 Table for the details of all the markers studied.

To identify the gene responsible for CSNB in this inbred pedigree with a common affected founder animal, the CSNB affected dogs are expected to be identical by descent for the causative mutation and flanking chromosomal segments. Hence we employed a strategy that is highly effective in inbred populations to identify recessive traits, and is particularly useful in dogs [53]. Using a combination of identity by descent and homozygosity mapping [54] we tested each candidate gene by haplotype analysis in phenotype-ascertained affected (1 male, 4 females) and carrier (3 males, 2 females) dogs using SNP and microsatellite repeat markers within and flanking the tested genes. Using this approach, we initially ruled out autosomal recessive cCSNB candidates *GRM6*, *TRPM1*, *GPR179* and *LRIT3*, as well as *GNB3*, *GNAT1*, *CACNA2D4*, *CABP4*, and *PDE6B*. In all cases, there was absence of a homozygous block of markers in the affecteds that was heterozygous in the carriers. Fig 4 illustrates the haplotypes with a subset of markers for the excluded genes in 5 representative animals in the pedigree. Additionally, for *LRIT3*, *GNB3* and *GNAT1*, all the exons as well as the exon-intron boundaries were sequenced to identify variants (pathogenic or polymorphic). No pathogenic mutation within the sequenced region was found.

Haplotype analysis could not be carried out for *RHO*, *NYX* and *CACNA1F*. All the markers selected from dbSNP as well as those identified following sequencing the exons and the exon-intron boundaries of *RHO* and *NYX* and their flanking regions were fixed in both the groups, presumably due to inbreeding. Absence of pathogenic mutation in the sequenced regions of *RHO* and *NYX* does not, however, rule out the possibility of the presence of a pathogenic variant in the promoter or deep within an intron resulting in change of gene expression or splicing that can result in disease. But since mutation in *RHO* causes the Riggs form of CSNB while *NYX* is X-linked we chose not to proceed further with these 2 genes. Similarly, for *CACNA1F* all the studied markers were uninformative in the pedigree. The coding region of this X-linked gene was not sequenced due to its large size and the autosomal recessive inheritance of the disease.

### Gene expression profiles in CSNB affected and carrier retinas

Quantitative RT-PCR was performed to study the expression pattern of those genes that are cone-specific (*OPS1LW*, *OPNISW* and *CNGB3*), rod-specific (*RHO*, *GNAT*, *PDE6B*, *CNGA1*, *CNGB1* and *SLC24A1*), expressed in Müller cells and astrocytes (*GFAP*), photoreceptor pre-synaptic terminal (*CACNA1F*, *CACNA2D4* and *CABP4*) and those that are either key elements (*GRM6*, *GNAO*, *GNB3*, *GPR179*, *TRPM1*, *NYX* and *LRIT3*) or regulators of mGluR6 cascade (*GNB5*, *PKCA*, *RGS11*, *RGS7BP* and *RGS9BP*). For this analysis, age-matched carrier (n = 2) and affected (n = 2) samples were compared to 3 normal controls. Because only 2 animals per disease genotype were included in the analyses, statistical analysis could not be performed.

**Fig 5:**
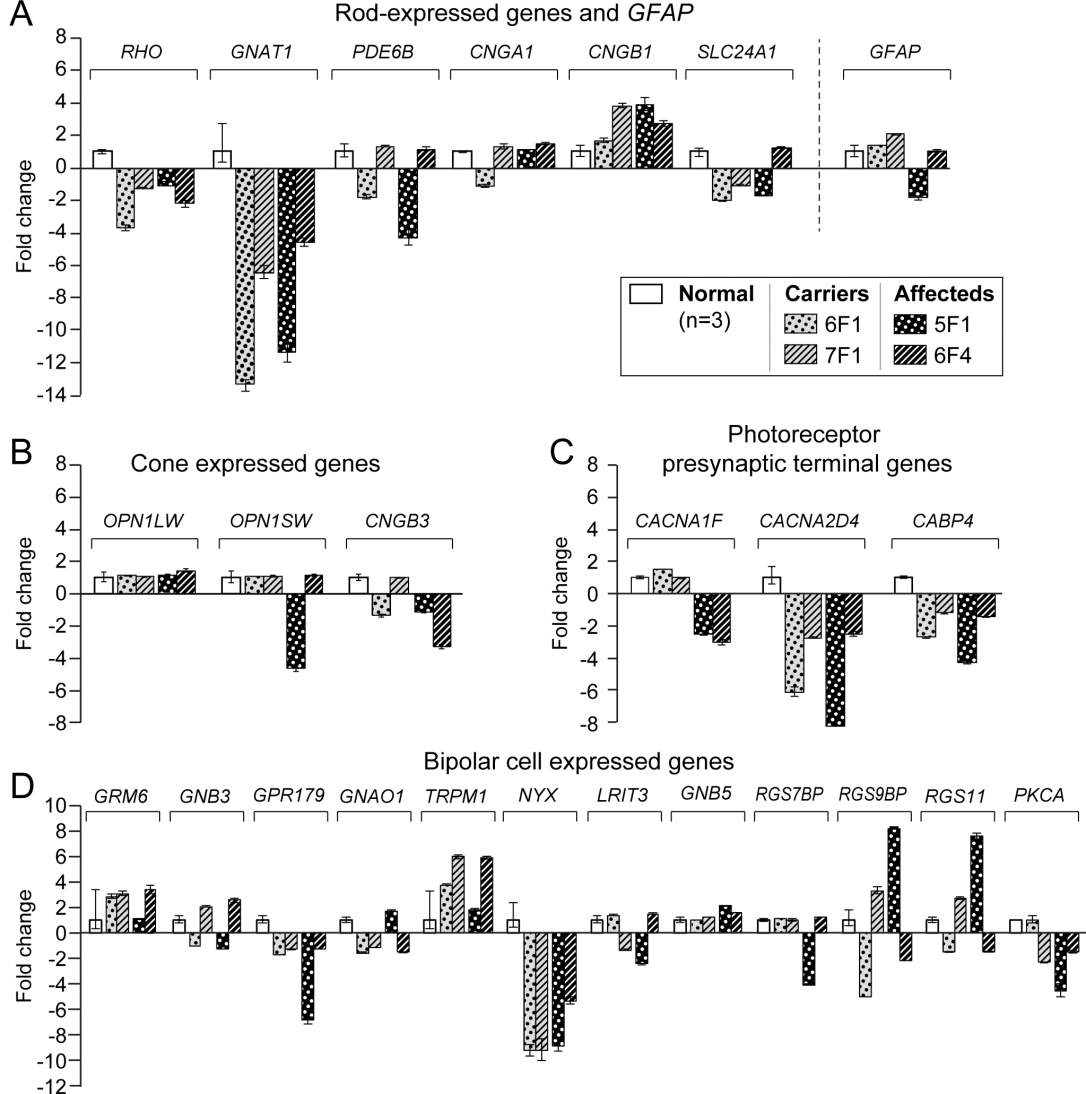
Gene expression profiles in CSNB affected and carrier retinas. Fold change differences measured by qRT-PCR of photoreceptor and CSNB candidate genes in 3 normal (mean ± SEM), and 2 each of carrier and affected (mean ± SD of 3 technical replicates) samples. **A**) Rod expressed genes and *GFAP*; **B**) cone expressed genes; **C**) photoreceptor presynaptic terminal genes; **D**) genes of mGluR6 cascade and modulators. Note that GNB3 is expressed both at the cone photoreceptors and bipolar cells whereas GNB5 in the rods.

We found inter animal gene expression variation that occurred between and within disease genotypes (Fig 5A–5D). Affected and carriers showed markedly decreased expression in *GNAT1*, *NYX and CACNA2D4*, while *CACNA1F* was down-regulated only in affecteds. Other genes, e.g. *PDE6B*, *CNGA1*, *OPN1LW*, *CNGB3*, *GNAO* and *RGS9BP* were variably expressed within groups, either in carriers or affecteds, and this difference was not age-related. As it is clear from Fig 5, the standard deviation derived from the 3 technical replicates of each sample represented less than 0.5–8.8% of the sample mean (except for *RHO*), an indication that the differences in gene expression levels in the different animals represented a biological, and not a technical variation.

### Normal photoreceptor structure and immunolabeling

**Fig 6:**
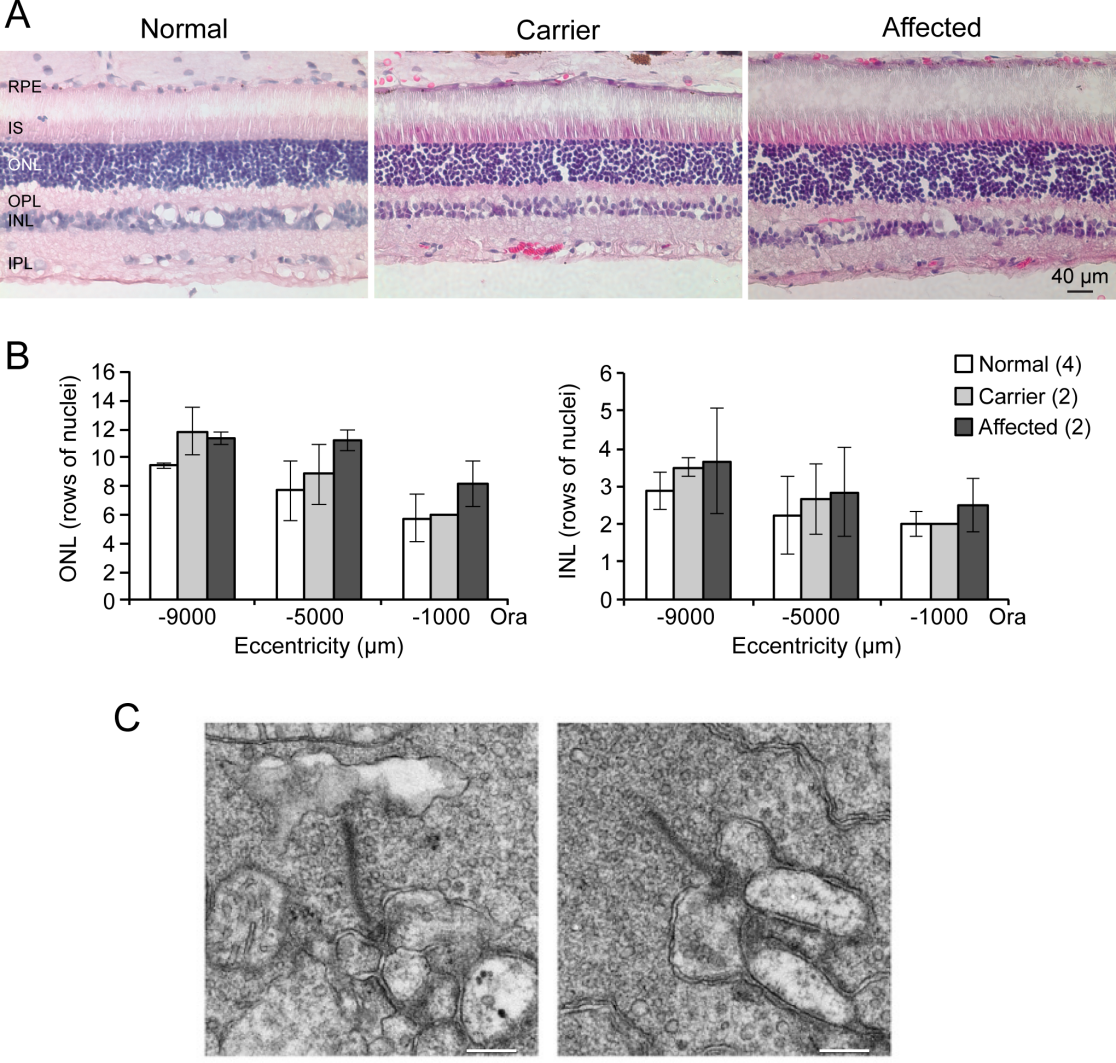
Normal retinal structure in CSNB affected dogs. **A**) Sections from normal, carrier and affected retinas stained with H&E show no structural abnormalities. The images are representative of four normal and two of each carrier and affected dogs. **B**) Graphical representations of outer (ONL) and inner (INL) nuclear layer thickness in the superior retina expressed as rows of nuclei. Number of nuclei was counted at positions 1000μm apart starting at 1000μm from the ora serrata in 4 normal, 2 carrier and 2 affected retinas. Only three representative positions are shown. **C**) Rod spherule ultrastructure of affected retina. The pre- and post-synaptic regions are normal. The bars indicate 200nm. RPE: retinal pigment epithelium; IS: inner segment; ONL: outer nuclear layer; OPL: outer plexiform layer; INL: inner nuclear layer.

Retinal morphology evaluated with H&E staining showed normal retinal structure in all genotypes (Fig 6A). Similarly, ONL and INL thickness measurements were comparable (Fig 6B). There was also no thinning of the retinal layers of the older carrier and affected dogs (7F1 and 6F4 respectively) relative to the younger carrier and affected ones (6F1 and 5F1 respectively) further supporting the non-progressive nature of the disease. At the ultra-structural level, the affected retina showed normal rod photoreceptor spherules with the classical structure of synaptic ribbons and vesicles, lateral horizontal cell processes and centrally placed invaginating rod bipolar dendrites. The post-synaptic elements invaginated into the rod terminal and formed triad or tetrad configuration adjacent to the ribbon site (Fig 6C).

**Fig 7:**
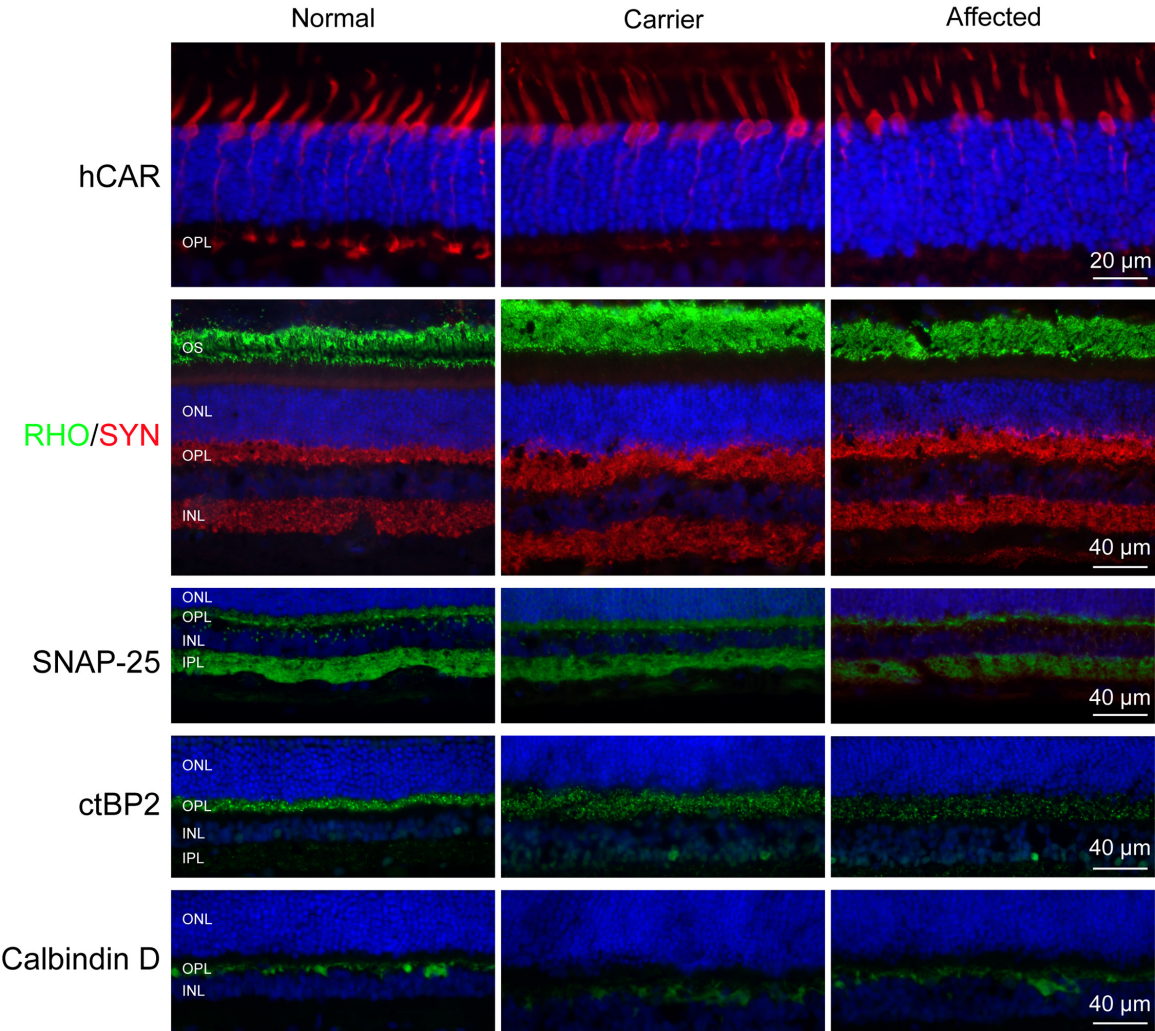
Outer retinal immunolabeling in normal, carrier and affected dogs. Cone arrestin, rod opsin and synaptophysin labeling are similar in the three genotypes. Punctate labeling of OPL synaptic terminals with SNAP-25 and CtBP2 was the same although the affected and carrier retinas showed more dispersed labeling interpreted as being a fixation artifact. Calbindin antibody clearly labels the horizontal cells in the scleral border of the INL. ONL: outer nuclear layer; OPL: outer plexiform layer; INL: inner nuclear layer, IPL: inner plexiform layer.

Since genes known to cause cCSNB disrupt the synaptic transmission from photoreceptors to depolarizing ON-bipolar cells, we used a battery of cell specific antibodies to assess the overall retinal integrity, and key cells in the pathway, and to localize the cellular defect. Labeling photoreceptors with hCAR (cones) and opsin (rods) antibodies showed normal immunolabeling pattern, and no rod opsin mislocalization (Fig 7). While cone arrestin labeling in the pedicles appeared less in affected and carrier retinas, this was not considered abnormal and is a variation frequently observed in normal. As cones contact both ON- and OFF-bipolar cells, and in cCSNB the ON-pathway is affected, the L/M- and S- cones also were labeled to see if there were differences in morphology or number, and none were found (data not shown). *GFAP* is one of the early genes up-regulated in retinal diseases and reflects an inner retinal stress response to outer retinal disease [55,56]. Single immunofluorescence labeling of GFAP indicated no difference in Müller cell and astrocyte structure and expression in the 3 groups (data not shown).

Synaptophysin showed normal labeling of terminals in the outer (OPL) and inner (IPL) plexiform layers, and indicated normal formation of synaptic vesicles at the OPL rod and cone photoreceptor terminals (Fig 7). To obtain an insight of the structure of the pre-synaptic terminals we then labeled the retinas with SNAP-25 and CtBP2 that label different photoreceptor terminals of the retina [57]. Distinct punctate labeling of SNAP-25 and CtBP2 was found in the OPL, and IPL (SNAP-25) in all the three groups although the intensity of the labeling decreased from the normal to carrier and then to affected (Fig 7). The dispersed pre-synaptic punctate labeling of both proteins likely represented fixation/processing artifacts. Lastly, examination of horizontal cells by calbindin immunolabeling showed no abnormalities (Fig 7).

### Altered expression of RGS11, Goα and PKCα in the mGluR6 cascade

**Fig 8:**
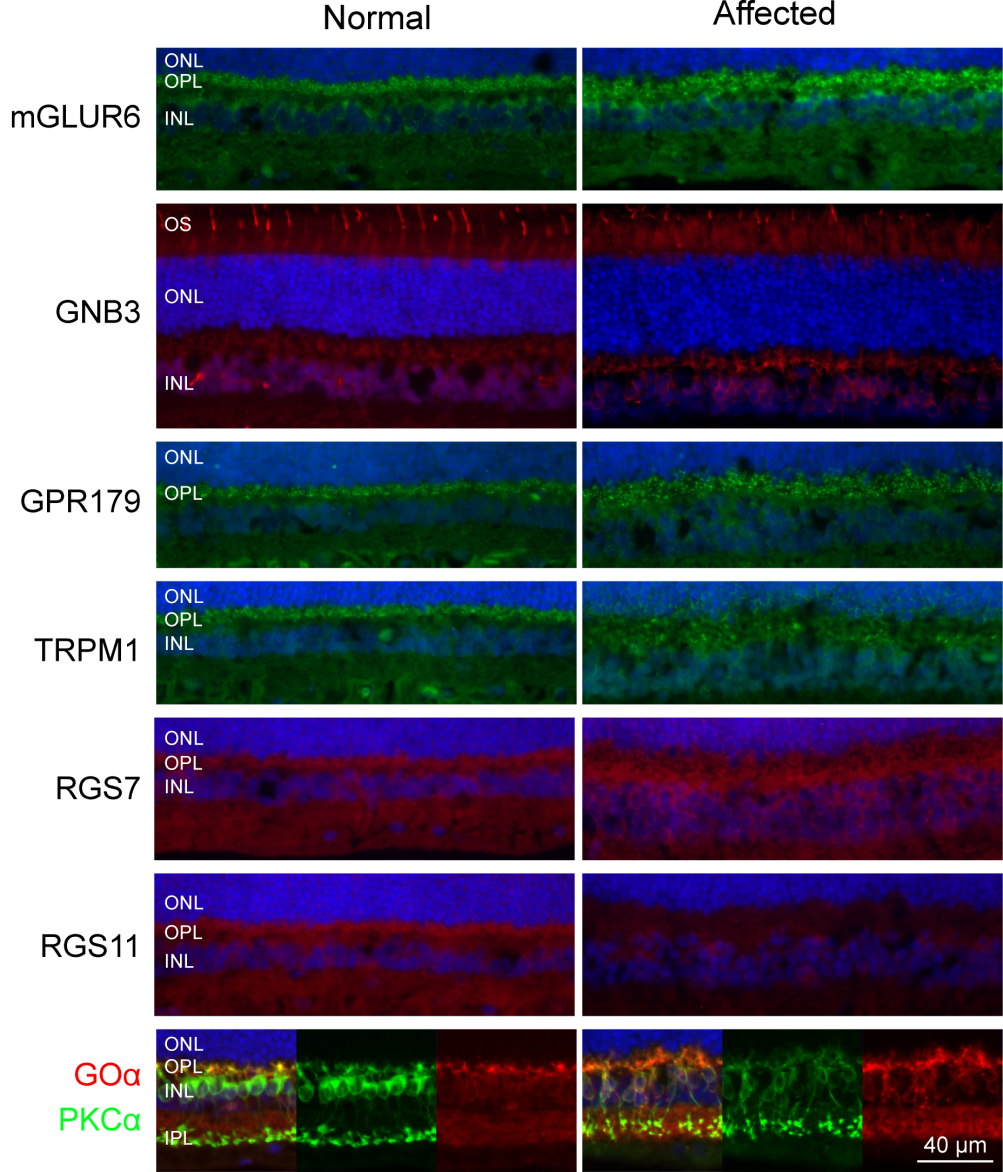
Immunofluorescence labeling of selected key proteins and modulators of mGluR6 cascade. mGluR6 showed punctate labeling at the tips of bipolar cells in the normal and affected retina. GNB3 only labeled the cone photoreceptors in normal and affected dogs; bipolar labeling was negligible as a result of the longer fixation time [71]. GPR179 and TRPM1 showed comparable labeling at the tips of bipolar cells in the normal and affected retinas. RGS7 labeling was comparable in bipolar cells of the normal and affected retinas. Normal retina labeled RGS11 but not the affected one. Goα labeling was increased in affected retina, and bipolar cell dendritic arbors extended into the OPL. PKCα labeling was decreased around the rod bipolar somata, and the axon terminals formed a thicker and intensely labeled layer in IPL. ONL: outer nuclear layer; OPL: outer plexiform layer; INL: inner nuclear layer, IPL: inner plexiform layer.

We next examined some of the major proteins involved in mGluR6 cascade since rods and cones communicate in this pathway through ON-bipolar cells. We found comparable labeling in the dendritic tips of BP cells between normal and affected retinas for mGluR6 (Fig 8), GNB3 which forms a complex with Goα to couple mGluR6 with TRPM1 channel in the ON-bipolar cells (Fig 8; [50]), RGS7, important in the development and function of the photoreceptor- bipolar cell synapse (Fig 8;[58]), GPR179, an interacting partner of TRPM1 (Fig 8), and TRPM1, the end product of the mGluR6 cascade (Fig 8). LRIT3 could not be evaluated as immunolabeling was weak and non-specific in all samples.

Abnormalities were found in the affected retina with RGS11, Goα and PKCα labeling. Both RGS7 and RGS11 are expressed at the tips of the BP cells and involved in the generation of ERG b-waves but in a functionally redundant manner [59]. Interestingly, RGS11 puncta were observable in the photoreceptor terminal side of the BP cells only in the normal, but not the affected (Fig 8). Goα, essential for the light response [36], is present in the ON-bipolar cells. PKCα, a modulator of the mGluR6 cascade, is expressed more or less uniformly in the RB cells. In the normal retina both antibodies colocalized in the BP cell dendritic arbors (Fig 8). PKCα also labeled the RB cell somata, and synaptic boutons in the IPL with equal intensity. In contrast, the affected retina had weakly labeled RB cells somata, and a broad and irregular layer of terminals rather than a discrete layer as in the normal. The Goα-labeled ON-bipolar cells showed more intense labeling in the affecteds. Double labeling showed dendritic projections extending towards ONL in the affected but not in the normal. The carrier retina showed an intermediate labeling pattern (data not shown).

## Discussion

Animal models have been instrumental in biomedical research for the identification of novel genes as well as informing on normal function and disease mechanisms. With respect to CSNB, mouse and horse models have led to the identification of *GRM6* [39], *GPR179* [22] and *TRPM1* [38,40,60,61] to be causal for cCSNB and *CABP4* [27] for icCSNB in humans. In dogs, at least 24 distinct forms of naturally-occurring retinal degeneration have been identified and characterized molecularly and all have initial defects in the photoreceptors or retinal pigment epithelium [62]. To date, there are no reports of CSNB in dogs; however, the disease currently known as canine LCA2 caused by a mutation in *RPE65* [63] was identified incorrectly in an early publication as a model of CSNB [64].

In the present study, we report a naturally occurring colony of beagle dogs where the affected animals were night blind but had normal day vision. These dogs had normal fundi and fluorescein angiograms. The full-field ERGs had a normal amplitude a-wave indicating that the phototransduction cascade was functioning normally. However, the ERG rod b-waves, which are believed to originate from rod ON-bipolar cells, were non-detectable. In addition, the photopic long-flash ERGs suggested a loss of the ON-responses, but preserved OFF-responses, indicating that the post-receptoral ON-pathway function is selectively affected. In addition, we confirmed that these functional abnormalities were stationary and not progressive. These clinical and functional characteristics of our affected dogs are very similar to those of humans with the complete-type CSNB of the Schubert-Bornschein form.

Because the disease is autosomal recessive, and the study population was small and highly inbred, we used homozygosity mapping to determine the association of known CSNB candidate genes with the disease. We did not find any significant co-segregation between the disease and the candidate gene markers of human autosomal recessive CSNB, viz., *GRM6*, *TRPM1*, *GPR179* and *LRIT3*. We also excluded candidate genes for other autosomal dominant and X-linked forms of CSNB, viz., *GNAT1*, *PDE6B*, *SLC24A1*, *CABP4*, as well as *GNB3* and *CACNA2D4*. Haplotype analysis could not be carried out for *RHO*, *NYX* and *CACNA1F*. *NYX* and *CACNA1F* are X-chromosomal and the studied disease is autosomal recessively inherited, while *RHO* causes the Riggs form of CSNB. Therefore the probability of any of these 3 genes to be causal to the disease is unlikely.

With one exception (*CACNA1F*), qRT-PCR expression profiles in affected and carrier retinas for genes whose products are expressed in rods, cones, photoreceptor presynaptic terminals, mGluR6 cascade genes and modulators, showed inter animal variability, and no consistent expression patterns that were disease-specific. The very low standard deviation of individual sample means suggested that the variation was biological, and not technical, and is a subject for future investigation. Within group variability in gene expression has been reported in a different disease model in dogs [48]. Interestingly, *CACNA1F* was the only gene that was down-regulated in affected animals but not in carriers and is likely a reflection of an ongoing disease process, but is not causal to the disease. The already ruled out genes, *GNAT1*, *NYX* and *CACNA2D4*, were down-regulated in both affected and carrier retinas; however, it is unknown at present whether decreased mRNA expression is accompanied by a low protein level. As to a functional significance of such decreases, it is important to note that heterozygous *GNAT1* knockout mice show ~50% reduction in rod transducin α protein, but the flash response of isolated rods were very similar to those of controls [65].

Retinal structure in mutant retinas was normal as were the synaptic ribbons and overall spherule structures. Counts of ONL and INL in carrier and affected of different ages showed no cell loss. By IHC, we found no differences from normal for antibodies labeling rods, cones and their presynaptic terminals. As well, GFAP showed lack of reactive Müller cells, an indication of absent outer retinal stress that is a characteristic response to progressive photoreceptor degenerations [55,56]. While we found no IHC abnormalities in selected members of the mGluR6 pathway or its modulators examined (mGluR6, GNB3, GPR179, RGS7 and TRPM1), we did find that RGS11, PKCα and Goα expression differed in affected vs control. Qualitatively, we found that PKCα showed decreased labeling in the rod BP somata and an intensely labeled broad layer of axon terminals in the affected whereas Goα had increased labeling in the dendritic terminals of ON-rod bipolars. Both markers showed dendritic extension of the ON-bipolar cells to the OPL whereas no labeling of RGS11 was observed in the mutant retina. These qualitative changes may not reflect the true decreases of protein expression as redistribution of the proteins within the cells may give the similar IHC appearance. However, they do point out to possible abnormalities in the signaling pathways examined.

ON-bipolar cells express mGluR6 which, when activated, couples with Goα and Gβγ subunits to close the TRPM1 channel, a critical step in the process of ON-bipolar cell signal transduction [50]. There is considerable debate over whether Gβγ dimer [66] or Goα [4] is responsible for the closure of the TRPM1 channel. Regardless, both these proteins are important for signaling in the ON-bipolar cells. *Gnao* ^-/-^ mice have low survival rates and fail to produce scotopic and photophic b-waves even though they have normal retinal morphology [4]. Interestingly, reduction in the expression of Goα does not change rod bipolar cell response [45]. Therefore we think change in Goα in our model might not cause a significant difference in the closure of TRPM1 channel. On the other hand, RGS11, required for the inactivation of Goα, labeled bipolar dendrites in the normal but not the affected retina. However, elimination of RGS11 is not enough to deactivate Goα to alter the ON-bipolar cell response to light [67] as other GTPase activating proteins can take up the function of inactivating Goα. Hence the absence of RGS11 labeling in the mutant retina is most likely to be a downstream effect in the disease pathogenesis. PKCα, required for the activation and termination of TRPM1 current [68], modulates synaptic transmission by relieving Mg^2+^ inhibition at the rod ON-bipolar cell synapse [69]. *Pkca-* ^/-^ mice have delayed but large amplitude rod b-wave (due to the fusion of the c-wave) and a normal cone response [68] that is a very different ERG pattern compared to our model. We therefore argue that the reduced expression of PKCα in the RB cells of affected dogs is not due to a mutation in *PKCA*. In fact, the change in Goα and PKCα is rather a secondary effect to a mutation in a yet unknown gene.

In conclusion, we have successfully established and characterized a canine pedigree of autosomal recessive cCSNB where the ON-bipolar cell function is compromised. We have excluded all known CSNB genes, an indication that another gene, likely localized in ON-bipolar cells, is involved. Identification of such a gene will provide further insights not only into signaling in ON-bipolar cells, likely in the mGluR6 pathway, but also serve as a novel candidate gene for CSNB in humans and animal models, and could be useful in testing new treatments including gene therapy or optogenetic manipulation in the future. Studies are ongoing to identify the causative gene by a whole genome approach, clarify the exact mechanism of night blindness and understand the neuronal plasticity and retinal remodeling that occurs in the disease.

## Supporting Information

S1 Movie Video of CSNB dog walking through an obstacle course under light and dim conditions. The video of an affected dog walking under dim illumination was recorded with an infrared camera. CSNB dogs could easily walk through the obstacle course in bright light conditions, but often collided with obstacles under the dim conditions. In the dark, they also walked slowly and raised their forelimbs (hypermetria) high to avoid collisions.
(MP4)

S1 Table Markers studied for haplotype analyses. Intragenic markers are in bold, and flanking markers in italics and orange. Markers that are fixed in the colony are italicized in the status column. For microsatellite repeat markers, the reference allele and the repeat markers (denoted by number) are separated by “/”. New variants identified in the study have been indicated as “Novel”. The coordinates of the genes and markers are based on CanFam2.0. Note that for *CACNA1F*, *RHO* and *NYX* the markers were fixed in the colony dogs, and exclusion of the gene from causal association with the disease by haplotype analysis could not be done.
(DOCX)

S2 Table Primers and assays for qRT-PCR. (DOCX)

S3 Table Antibodies used for immunohistochemistry. (DOCX)
