# Supplementary material for: A Naturally Occurring Canine Model of Autosomal Recessive Congenital Stationary Night Blindness
Source: PLoS One. 2015 Sep 14;10(9):e0137072. doi: 10.1371/journal.pone.0137072 (PMC4569341; doi:10.1371/journal.pone.0137072)
Supplement: S1 Table — Intragenic markers are in bold, and flanking markers in italics and orange. Markers that are fixed in the colony are italicized in the status column. For microsatellite repeat markers, the reference allele and the repeat markers (denoted by number) are separated by “/”. New variants identified in the study have been indicated as “Novel”. The coordinates of the genes and markers are based on CanFam2.0. Note that for CACNA1F, RHO and NYX the markers were fixed in the colony dogs, and exclusion of the gene from causal association with the disease by haplotype analysis could not be done. (DOCX) [file pone.0137072.s002.docx]

**S1 Table. Makers used for haplotype analysis.**

|  | **GENE** | | **CFA2.0 POSITION** | **REF ALLELE** | **STATUS** |
| --- | --- | --- | --- | --- | --- |
| 1 | ***RHO* (5254bp)** | | **Chr20: 8636212-8641466** |  |  |
|  |  | | *Chr20:8635017* | G | *rs22866471* |
|  |  | | **Chr20:8636791** | **C** | *rs22867294* |
|  |  | | **Chr20:8637761** | **T** | *Novel* |
|  |  | | **Chr20:8637807** | **C** | *Novel* |
|  |  | | **Chr20:8637808** | **C** | *Novel* |
|  |  | | **Chr20:8638174** | **G** | *rs22867297* |
|  |  | | **Chr20:8638411** | **A** | *Novel* |
|  |  | | **Chr20:8639516** | **G** | *rs22881035* |
|  |  | | **Chr20:8640879** | **C** | *rs8884153* |
|  |  | | *Chr20:8657973* | G | *rs22852957* |
| 2 | ***GNAT1* (3889bp)** | | **Chr20:42129361-42133250:-1** |  |  |
|  |  | | *Chr20:41745091* | C | rs22848857 |
|  |  | | **Chr20:42129407** | **A** | *Novel* |
|  |  | | **Chr20:42130351** | **T** | *Novel* |
|  |  | | **Chr20:42130354** | **T** | *Novel* |
|  |  | | **Chr20:42131456** | **C** | Novel |
|  |  | | **chr20:42131682** | **C** | Novel |
|  |  | | **Chr20:42131909** | **G** | Novel |
|  |  | | *Chr20:42272764* | A | rs8679039 |
| 3 | ***PDE6B* (28110bp)** | **Chr3:94,573,287-94,601,397 :-1** | |  |  |
|  |  | *Chr3:94526955* | | G | rs23644564 |
|  |  | **Chr3:94574787** | | **G** | *rs23644611* |
|  |  | **Chr3:94574810** | | **T** | rs23570494 |
|  |  | **Chr3:94578249** | | **T** | rs23644612 |
|  |  | **Chr3:94588307** | | **T** | *rs23644616* |
|  |  | **Chr3:94601047** | | **C** | Novel |
|  |  | **Chr3:94601131** | | **T** | Novel |
|  |  | *Chr3:94622240* | | A | rs23644637 |

|  | **GENE** | **CFA2.0 POSITION** | **REF ALLELE** | **STATUS** |
| --- | --- | --- | --- | --- |
| 4 | ***SLC24A1* (23728bp)** | **Chr30:32962460-32986188:1** |  |  |
|  |  | *Chr30:32941089* | C | rs23649011 |
|  |  | **Chr30:32981210** | **G** | *Novel* |
|  |  | **Chr30:32981344** | **A** | *rs23618221* |
|  |  | **Chr30:32983855** | **T** | rs9189260 |
|  |  | **Chr30:32985975** | **G** | *Novel* |
|  |  | **Chr30:32986002** | **C** | Novel |
|  |  | **Chr30:32986036** | **G** | Novel |
|  |  | **Chr30:32986051** | **G** | *Novel* |
|  |  | **Chr30:32986101** | **C** | Novel |
|  |  | **Chr30:32986115** | **T** | Novel |
|  |  | **Chr30:32986205** | **G** | *Novel* |
|  |  | *Chr30:32994130* | G | rs23654822 |
| 5 | ***CACNA1F* (24507bp)** | **ChrX:42256561-42281312:-1** |  |  |
|  |  | *ChrX:42206541* | A | *rs24648953* |
|  |  | **ChrX:42262363** | **T** | *Novel* |
|  |  | **ChrX:42262471** | **G** | *Novel* |
|  |  | **ChrX:42272395** | **G** | *rs397513541* |
|  |  | **ChrX:42272414** | **T** | *rs397510750* |
|  |  | **ChrX:42272461** | **C** | *rs397512587* |
|  |  | **ChrX:42277744** | **G** | *Novel* |
|  |  | **ChrX:42277759** | **G** | *Novel* |
|  |  | **ChrX:42279335** | **C** | *Novel* |
|  |  | **ChrX:42279344** | **T** | *Novel* |
|  |  | **ChrX:42279356** | **A** | *Novel* |
|  |  | **ChrX:42279367** | **C** | *Novel* |
|  |  | **ChrX:42279371** | **T** | *Novel* |
|  |  | **ChrX:42279424** | **A** | *Novel* |
|  |  | *ChrX:42356040* | T | *rs24635274* |
| 6 | ***CACNA2D4* (70213bp)** | **Chr27:46923095-46993308:-1** |  |  |
|  |  | *Chr27:46919853* | G | rs9120280 |
|  |  | **Chr27:46925581** | **G** | rs8978344 |
|  |  | **Chr27:46937382** | **T** | rs9074957 |
|  |  | **Chr27:46958211** | **G** | rs8557390 |
|  |  | **Chr27:46960825** | **T** | Novel |
|  |  | **Chr27:46971876** | **C** | Novel |
|  |  | **Chr27:46986948** | **G** | Novel |
|  |  | **Chr27:46987436** | **A** | rs23364247 |
|  |  | *Chr27:47016481* | C | rs23354672 |

|  | **GENE** | **CFA2.0 POSITION** | **REF ALLELE** | **STATUS** |
| --- | --- | --- | --- | --- |
| 7 | ***CABP4* (3156bp)** | **Chr18: 53040790-53043946** |  |  |
|  |  | *Chr18:53034831* | A | rs22662219 |
|  |  | **Chr18:53041662** | **C** | Novel |
|  |  | **Chr18:53041698** | **G** | Novel |
|  |  | **Chr18:53042536** | **G** | Novel |
|  |  | **Chr18:53042688** | **T** | Novel |
|  |  | **Chr18:53043559** | **C** | Novel |
|  |  | **Chr18:53043596** | **A** | rs22657110 |
|  |  | *Chr18:53056183* | G | rs22629739 |
| 8 | ***GRM6* (20799bp)** | **Chr11:5580160-5600959:1** |  |  |
|  |  | *Chr11:5525910* | A | rs22119976 |
|  |  | **Chr11:5592980** | **G** | Novel |
|  |  | **Chr11:5593182** | **A** | Novel |
|  |  | **Chr11:5592738** | **C** | *rs8971374* |
|  |  | **Chr11:5598326** | **G/15** | Novel |
|  |  | *Chr11:5614265* | C | rs22069104 |
| 9 | ***GNB3* (23443bp)** | **Chr27:41204299-41227742:-1** |  |  |
|  |  | *Chr27:41157931* | C | rs23373704 |
|  |  | **Chr27:41207183** | **G** | *rs23340955* |
|  |  | **Chr27:41207340** | **C** | rs23340954 |
|  |  | **Chr27:41209158** | **T** | *Novel* |
|  |  | **Chr27:41224027** | **G** | rs23341433 |
|  |  | *Chr27:41321348* |  | rs23365057 |
| 10 | ***GPR179* (15229bp)** | **Chr9: 27,136,393-27,151,622:1** |  |  |
|  |  | *Chr9:27085898* | A | rs24604622 |
|  |  | **Chr9:27138303** | **G** | Novel |
|  |  | **Chr9:27141056** | **A** | *rs24604654* |
|  |  | **Chr9:27142163** | **T/15** | Novel |
|  |  | **Chr9:27143339** | **A** | *Novel* |
|  |  | **Chr9:27143412** | **T** | *Novel* |
|  |  | **Chr9:27143416** | **T** | *Novel* |
|  |  | **Chr9:27144527** | **C** | *rs24556456* |
|  |  | **Chr9:27148212** | **G** | Novel |
|  |  | **Chr9:27148827** | **G** | Novel |
|  |  | **Chr9:27148925** | **G** | Novel |
|  |  | *Chr9:27168219* | A | rs8770636 |

|  | | | **GENE** | | | **CFA2.0 POSITION** | **REF ALLELE** | **STATUS** | |
| --- | --- | --- | --- | --- | --- | --- | --- | --- | --- |
| 11 | | | ***TRPM1* (132001bp)** | | | **Chr3:40,672,026-40,804,027:1** |  |  | |
|  | | |  | | | *Chr3:40647962* | C | rs23584437 | |
|  | | |  | | | **Chr3:40690909** | **T** | rs23606583 | |
|  | | |  | | | **Chr3:40704476** | **C** | rs8974936 | |
|  | | |  | | | **Chr3:40734502** | **C** | rs23602170 | |
|  | | |  | | | **Chr3:40734806** | **A** | rs23579590 | |
|  | | |  | | | **Chr3:40741670** | **A** | rs23579613 | |
|  | | |  | | | **Chr3:40741695** | **G** | Novel | |
|  | | |  | | | **Chr3:40741743** | **G** | Novel | |
|  | | |  | | | **Chr3:40782544** | **A** | rs23579749 | |
|  | | |  | | | **Chr3:40790580** | **A** | rs23579824 | |
|  | | |  | | | **Chr3:40795580** | **T** | rs23504997 | |
|  | | |  | | | **Chr3:40803105** | **G** | rs23573538 | |
|  | | |  | | | **Chr3:40803305** | **A** | rs23573540 | |
|  | | |  | | | **Chr3:40803359** | **C** | rs23573542 | |
|  | | |  | | | **Chr3:40804943** | **G** | rs23596973 | |
|  | | |  | | | *Chr3:40844380* | A | rs23579107 | |
| 12 | | ***NYX* (18713bp)** | | | **ChrX:35816030-35834743:1** | |  |  | |
|  | |  | | | *ChrX:35805198* | | C | *rs24624616* | |
|  | |  | | | **ChrX:35816631** | | **T** | *Novel* | |
|  | |  | | | **ChrX:35816639** | | **A** | *Novel* | |
|  | |  | | | **ChrX:35818999** | | **T** | *Novel* | |
|  | |  | | | **ChrX:35819117** | | **A** | *Novel* | |
|  | |  | | | **ChrX:35835023** | | **T** | *Novel* | |
|  | |  | | | **ChrX:35836479** | | **A** | *rs24628079* | |
|  | |  | | | **ChrX:35836787** | | **T** | *rs24589868* | |
|  | |  | | | **ChrX:35836796** | | **T** | *rs24589869* | |
|  | |  | | | *ChrX:35837065* | | C | *rs24609756* | |
| 13 | ***LRIT3* (15084bp)** | | | **Chr32: 32947384-32962468:1** | | |  | |  |
|  |  | | | *Chr32:32959173* | | | G | rs23780832 | |
|  |  | | | **Chr32:32948905** | | | **T** | *rs23746705* | |
|  |  | | | **Chr32:32951642** | | | **C** | rs23756706 | |
|  |  | | | **Chr32:32951656** | | | **T** | rs23756709 | |
|  |  | | | **Chr32:32954614** | | | **A** | rs8570931 | |
|  |  | | | **Chr32:32958264** | | | **T** | Novel | |
|  |  | | | **Chr32:32958594** | | | **T** | rs23784477 | |
|  |  | | | **Chr32:32958958** | | | **T** | Novel | |
|  |  | | | **Chr32:32961693** | | | **C** | Novel | |
|  |  | | | **Chr32:32962372** | | | **C** | rs23754540 | |
|  |  | | | **Chr32:32962786** | | | **C** | rs23776597 | |
|  |  | | | **Chr32:32962880** | | | **C** | rs23783964 | |
|  |  | | | *Chr32:32985264* | | | G | rs8929808 | |

Markers in **bold** are intragenic, and those in *italics and orange* are flanking markers.
