# Supplementary material for: A Naturally Occurring Canine Model of Autosomal Recessive Congenital Stationary Night Blindness
Source: PLoS One. 2015 Sep 14;10(9):e0137072. doi: 10.1371/journal.pone.0137072 (PMC4569341; doi:10.1371/journal.pone.0137072)
Supplement: S2 Table — (DOCX) [file pone.0137072.s003.docx]

**S2 Table: Primers and Assays for qRT-PCR.**

1. **Primers for SYBR Green Assay**

| **Gene** | **Forward primer** | **Reverse primer** | **Product size (bp)** |
| --- | --- | --- | --- |
| *GFAP* | ACTTGCAGATCCGAGAAAC | CTCCACGGTCTTTACCACAAT | 86 |
| *RHO* | ATGATTGTCATATTCTTCTGCTATGGA | TGGGTGGTGGCCGATTC | 86 |
| *GNAT1* | GGATGCTCGAACCGTGAAAC | GCAATGAACTCGAGGCACTCTT | 122 |
| *PDE6B* | CTGAACTTCGGCACCTTAAACC | CTCGATGTCCGTCAGCTCCT | 126 |
| *SLC24A1* | TGGTGGTCAGGAAGGAGAG | CACCTCCACCTTGTCCATTT | 104 |
| *CNGB3* | GACTCCAGTCACGTTTGAAGA | GGATGTTGATGGTTGGGATTTG | 102 |
| *CACNA1F* | GGACATTCATCAAGTCCTTCCA | CCTTGCCAAACATCTGCATTC | 102 |
| *CACNA2D4* | GCAGCTCCACTGGATTCTTCA | AGCTTGTATGTACCAGCCACGA | 107 |
| *CABP4* | TCATCGAGGTCTCCCAGCAC | CCCTGAGCTTTGGGCTCATC | 90 |
| *GRM6* | TGCATCATCTGGCTGGCTTT | GACGCACTCAGGCTCAAGGA | 110 |
| *GNB3* | GGAGCTACACCTCAACCTATCA | CTTTCCTGGCATCTGCAATCT | 95 |
| *GPR179* | AGGCTCCTACCTGGACAGCA | CGGCCATTTCCTTGGTCTT | 128 |
| *TRPM1* | GATGAGCAGCAGTCGTCCAG | TTTCTCTTTCGTTGATTTCTTCCA | 97 |
| *NYX* | CACAGCTGGGTGGTTCTAAG | CCAAGTAGGGAACAATGTCGAT | 103 |
| *LRIT3* | AACCACACTACCACCAGATTTCC | TCACAGAACCAAGGGTTGTCC | 129 |

1. **Primers for TaqMan Assay**

| **Gene** | **Forward primer** | **MGB Probe** | **Reverse primer** | **Product size (bp)** |
| --- | --- | --- | --- | --- |
| *OPS1LW* | CAGCGTCATCATACTGTGCTACCT | ATCCGAGCGGTGGC | GGACTCAGATTCTTTCTGCTGCTT | 79 |
| *OPN1SW* | CGCCATGTTTGTGCTTTGG | CTCTACAGCAGGTCTGGTGA | GATGACAATGTAGCGCTCAAAGG | 99 |

1. **TaqMan Gene Expression Assays**

| **Gene** | **Assay#** | **Product size (bp)** |
| --- | --- | --- |
| *GAPDH* | Hs02786624_g1 | 157 |
| *CNGB1* | Cf02662052_m1 | 124 |
| *CNGA1* | Cf02628568_m1 | 98 |
| *GNB5* | Cf02631024_m1 | 92 |
| *RGS7BP* | Cf00936133_m1 | 110 |
| *RGS9BP* | Cf02678958_s1 | 123 |
| *RGS11* | Cf02660333_m1 | 66 |
| *PKCA* | Cf02655322_m1 | 97 |
