# Supplementary material for: A Naturally Occurring Canine Model of Autosomal Recessive Congenital Stationary Night Blindness
Source: PLoS One. 2015 Sep 14;10(9):e0137072. doi: 10.1371/journal.pone.0137072 (PMC4569341; doi:10.1371/journal.pone.0137072)
Supplement: S3 Table — (DOCX) [file pone.0137072.s004.docx]

**S3 Table. Antibodies used for** **immunohistochemistry**

| **Marker** | **Host** | **Normal retinal localization or target protein** | **Working dilution** | **Source, Catalog # or Name** |
| --- | --- | --- | --- | --- |
| **Photoreceptors** | | | | |
| **Rhodopsin** | mouse monoclonal IgG1 | OS of rods [[1](#_ENREF_1)] | 1:1000 | Millipore, MAB5316 |
| **hCAR** | goat polyclonal IgG | Red, green and blue sensitive cones [[2](#_ENREF_2)] | 1:1000; * | Custom made W. Beltran |
| **L/M cone opsin** | Rabbit IgG polyclonal | OS of L/M cone [[3](#_ENREF_3)] | 1:1000; * | Millipore, AB 5405 |
| **S cone opsin** | Rabbit IgG polyclonal | OS of S-cone [[3](#_ENREF_3)] | 1:1000; * | Millipore, AB 5407 |
| **Synapse** | | | | |
| **Synaptophysin** | rabbit polyclonal | Photoreceptor terminal [[4](#_ENREF_4)] | 1:100 | Dako Cytomation, A0010 |
| **CtBP2** | mouse monoclonal IgG1 | ribbons in photoreceptor and bipolar cell terminals [[5](#_ENREF_5)] | 1:2,000 | BD Biosciences, 612044 |
| **SNAP-25** | mouse monoclonal IgG1 | Base of OS and terminal regions of photoreceptors; cholinergic amacrine cells [[6](#_ENREF_6)] | 1:5,000; * | Millipore, MAB331 |
| **Inner retina and glia** | | | | |
| **GOα** | mouse monoclonal IgG1 | ON- (rod and cone) bipolar  cells [[7](#_ENREF_7)] | 1:5,000; * | Millipore, MAB3073 |
| **PKCα** | mouse monoclonal IgG2b | Rod bipolar and to a lesser extent in amacrine cells and cone bipolar cells [[8](#_ENREF_8),[9](#_ENREF_9)] | 1:100; * | BD Transduction Laboratories, 610107 |
| **GNB3** | rabbit polyclonal IgG | Islet-1 positive cone ON bipolar cells and cone photoreceptors [[10](#_ENREF_10)] | 1:300 | Santa Cruz Biotechnology, Inc, SC-381 |
| **mGLUR6** | rabbit polyclonal | ON- bipolar cells [[11](#_ENREF_11)] | 1:500; * | N. Vardi |
| **TRPM1** | rabbit polyclonal | ON- bipolar dendrites that invaginate photoreceptor terminals and synaptic ribbons of a subclass of rod [[12](#_ENREF_12)] | 1:100; * | Sigma, HPA014785 |
| **GPR179** | rabbit polyclonal | Tips of ON- bipolar cells [[13](#_ENREF_13)] | 1:1000; * | Sigma, HPA017885 |
| **LRIT3** | rabbit polyclonal | Dendrites of depolarizing bipolar cells [[14](#_ENREF_14)] | no specific labeling on canine retinal sections | Sigma, HPA013454 |
|  | mouse polyclonal |  | no specific labeling on canine retinal sections | Abnova, H00345193-B01P |
| **GFAP** | rabbit polyclonal IgG | Müller cells and astrocytes [[15](#_ENREF_15)] | 1:1000 | Dako, Z0334 |
| **Calbindin D-28K** | rabbit polyclonal | Horizontal and amacrine cells [[16](#_ENREF_16)] | 1:1000; * | Sigma, C2724 |

*Antigen retrieval.

CtBP2: C-terminal-Binding Protein 2; GNB3: guanine nucleotide-binding protein β3; GPR179: G protein receptor; GFAP: Glial fibrillary acidic protein; hCAR: Human Cone arrestin; LRIT3: leucine-rich-repeat (LRR) immunoglobulin-like and transmembrane-domain 3 ; mGLUR6: metabotropic glutamate receptor 6; OS: outer segment; PKCα: Protein kinase C; SNAP-25: Synaptosomal-associated protein 25; TRPM1: Transient receptor potential cation channel, subfamily M, member 1.

**Reference**

1. Adamus G, Zam ZS, Arendt A, Palczewski K, McDowell JH, et al. (1991) Anti-rhodopsin monoclonal antibodies of defined specificity: characterization and application. Vision Res 31: 17-31.

2. Sakuma H, Inana G, Murakami A, Higashide T, McLaren MJ (1996) Immunolocalization of X-arrestin in human cone photoreceptors. FEBS Lett 382: 105-110.

3. Zhang Q, Beltran WA, Mao Z, Li K, Johnson JL, et al. (2003) Comparative analysis and expression of CLUL1, a cone photoreceptor-specific gene. Invest Ophthalmol Vis Sci 44: 4542-4549.

4. Brandstatter JH, Lohrke S, Morgans CW, Wassle H (1996) Distributions of two homologous synaptic vesicle proteins, synaptoporin and synaptophysin, in the mammalian retina. J Comp Neurol 370: 1-10.

5. tom Dieck S, Altrock WD, Kessels MM, Qualmann B, Regus H, et al. (2005) Molecular dissection of the photoreceptor ribbon synapse: physical interaction of Bassoon and RIBEYE is essential for the assembly of the ribbon complex. J Cell Biol 168: 825-836.

6. Greenlee MH, Roosevelt CB, Sakaguchi DS (2001) Differential localization of SNARE complex proteins SNAP-25, syntaxin, and VAMP during development of the mammalian retina. J Comp Neurol 430: 306-320.

7. Vardi N (1998) Alpha subunit of Go localizes in the dendritic tips of ON bipolar cells. J Comp Neurol 395: 43-52.

8. Fyk-Kolodziej B, Cai W, Pourcho RG (2002) Distribution of protein kinase C isoforms in the cat retina. Vis Neurosci 19: 549-562.

9. Ruether K, Feigenspan A, Pirngruber J, Leitges M, Baehr W, et al. (2010) PKC{alpha} is essential for the proper activation and termination of rod bipolar cell response. Invest Ophthalmol Vis Sci 51: 6051-6058.

10. Ritchey ER, Bongini RE, Code KA, Zelinka C, Petersen-Jones S, et al. (2010) The pattern of expression of guanine nucleotide-binding protein beta3 in the retina is conserved across vertebrate species. Neuroscience 169: 1376-1391.

11. Vardi N, Morigiwa K, Wang TL, Shi YJ, Sterling P (1998) Neurochemistry of the mammalian cone 'synaptic complex'. Vision Res 38: 1359-1369.

12. Klooster J, Blokker J, Ten Brink JB, Unmehopa U, Fluiter K, et al. (2011) Ultrastructural localization and expression of TRPM1 in the human retina. Invest Ophthalmol Vis Sci 52: 8356-8362.

13. Klooster J, van Genderen MM, Yu M, Florijn RJ, Riemslag FC, et al. (2013) Ultrastructural localization of GPR179 and the impact of mutant forms on retinal function in CSNB1 patients and a mouse model. Invest Ophthalmol Vis Sci 54: 6973-6981.

14. Zeitz C, Jacobson SG, Hamel CP, Bujakowska K, Neuille M, et al. (2013) Whole-exome sequencing identifies LRIT3 mutations as a cause of autosomal-recessive complete congenital stationary night blindness. Am J Hum Genet 92: 67-75.

15. John SK, Smith JE, Aguirre GD, Milam AH (2000) Loss of cone molecular markers in rhodopsin-mutant human retinas with retinitis pigmentosa. Mol Vis 6: 204-215.

16. Hamano K, Kiyama H, Emson PC, Manabe R, Nakauchi M, et al. (1990) Localization of two calcium binding proteins, calbindin (28 kD) and parvalbumin (12 kD), in the vertebrate retina. J Comp Neurol 302: 417-424.
